# Knowledge, Attitude, Practice, and Perceived Barriers Regarding Colorectal Cancer Screening Practices Among Healthcare Practitioners: A Systematic Review

**DOI:** 10.7759/cureus.54381

**Published:** 2024-02-17

**Authors:** Majdi M Alzoubi, Suhair H Al-Ghabeesh

**Affiliations:** 1 Department of Nursing, Al-Zaytoonah University, Amman, JOR

**Keywords:** attitude, knowledge, perceived barriers, healthcare practitioners, colorectal cancer

## Abstract

The recommendations of medical professionals play a significant role in colorectal cancer (CRC) screening. This study aims to systematically review knowledge, attitude, practice, and perceived barriers regarding CRC screening practices among healthcare practitioners (HCPs). From January 2023 to December 2023, a comprehensive literature search was conducted using online databases, including Web of Science, PubMed, Scopus, and Research Gate, by using the following keywords in combination: "knowledge," "attitude," "practice," "perceived barriers," "colorectal cancer," and "health practitioners." The researchers screened and examined the retrieved literature. A total of 21 studies were considered relevant for the current review. Among these studies, eight assessed the level of knowledge, attitude, practices, and perceived barriers toward CRC screening among various health practitioners. Three studies assessed knowledge and attitudes toward CRC screening among health practitioners. The remaining ten studies assessed awareness, perceived barriers, or only knowledge of CRC screening among HCPs. In addition, all the included studies employed a cross-sectional design. The review shows that many healthcare providers need more fundamental knowledge of CRC screening. Healthcare procedures must be improved to enhance the knowledge, attitudes, and practices of healthcare professionals regarding CRC screening and their understanding of the associated barriers.

## Introduction and background

Colorectal cancer (CRC) is one of the leading causes of cancer-related deaths in both industrialized and developing nations [1]. Researchers predict that the global male age-standardized CRC rates will be 20.6/100,000 and the female age-standardized rates will be 14.3/100,000 [1]. In 2012, CRC diagnoses reached 1.4 million, leading to 693,900 deaths [2]. According to global cancer projections, the Middle East and Western Asia are seeing rising rates of CRC, which is due mainly to the rising incidence of CRC risk factors [2].

Current evidence establishes age as a significant contributing factor to the occurrence of CRC, with the risk increasing at the age of 40; after the age of 50, the risk of CRC diagnosis significantly increases [3]. Other risk factors, such as alcohol, smoking, familial or genetic polyposis, and ulcerative colitis, are also associated with an increased risk of CRC [4]. Screening procedures in primary healthcare settings, where primary healthcare physicians play a significant role in the early detection of cancers and chronic disorders, are part of CRC prevention methods [4, 5]. Furthermore, experts recommend improving current medical education, starting at the academic degree level, for prevention and early identification [4].

Early identification and removal of precancerous polyps avoid the earliest stage of CRC pathogenesis [4]. A study conducted by Zauber et al. stated that identifying and excising colon polyps in their early stages can avoid CRC mortality [6]. Most worldwide guidelines suggest screening individuals 50-75 years of age for CRC every 10 years with a colonoscopy, every five years with a flexible sigmoidoscopy, or every year with a fecal occult blood test (FOBT) [7]. Therefore, primary care physicians and healthcare practitioners (HCPs) are essential in the early identification of cancer. Research has indicated that general practitioners diagnose most cancer patients at primary care centers [7].

Several studies have shown that enhancing HCPs' knowledge and attitude about CRC screening enables them to adhere more closely to screening recommendations and ensure proper administration of tests [7]. The HCPs' backgrounds also have a significant role as they influence their capacity to offer CRC screening services. HCPs with personal experience with CRC, such as having a family member with the disease or treating a patient with it, are more likely to offer or suggest CRC screening to all eligible patients [7]. Therefore, we aimed to conduct a systematic review on assessing knowledge, attitude, and perceived barriers regarding CRC screening practices among HCPs.

Method

Search Strategy

This review utilized the Preferred Reporting Items for Systematic Review and Meta-Analysis Protocols (PRISMA-P) search strategies. The researchers completed a literature search for relevant studies using the electronic databases PubMed, Science Direct, Scopus, Research Gate, and Google Scholar. The search strategy consisted of keywords and controlled vocabulary terms related to CRC, knowledge, attitude, perceived barriers, and HCPs. In order to conduct a comprehensive search, we also explored additional sources, including reference lists of relevant articles and grey literature.

Inclusion and Exclusion Criteria

The inclusion criteria for articles used in this review were 1) studies conducted among healthcare professionals regardless of ethnicity, country, or profession, 2) experimental studies that assessed knowledge, attitude, and perceived barriers, 3) studies related to CRC, and 4) articles published in English. Exclusion criteria were narrative reviews and studies conducted among non-healthcare professionals. In addition, this review excluded dissertations, theses, monographs, and commentaries.

Data Extraction

Two reviewers independently performed data extraction using a standardized form. The reviewers extracted participant characteristics (sample size, gender, age, and profession), study design, country of study, follow-up period (weeks or months) for assessing knowledge, attitude, and practice regarding CRC, and study outcome details from the retrieved studies.

Quality Assessment

The authors assessed the methodological quality and risk of bias in the included studies using the Cochrane Risk of Bias tool.

Results

**Figure 1:**
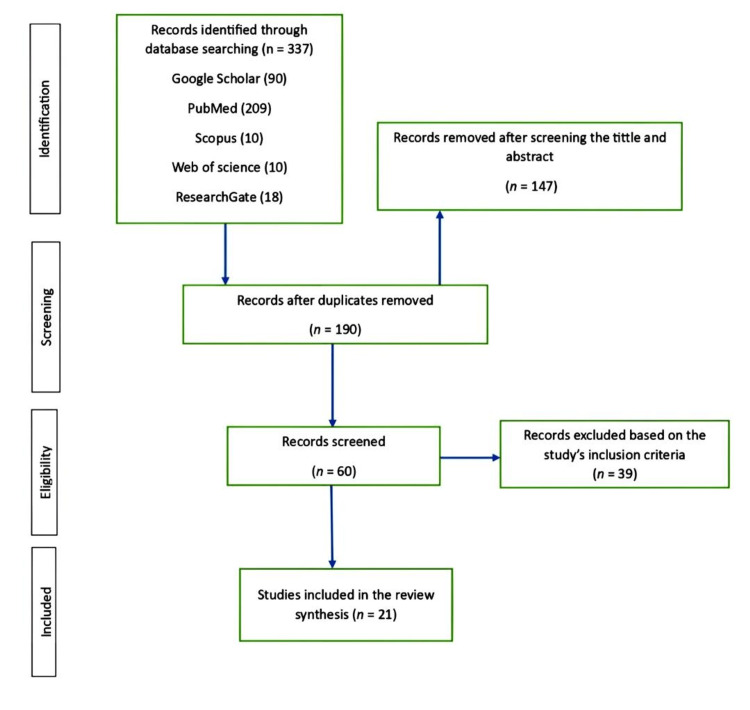
PRISMA flow diagram PRISMA, Preferred Reporting Items for Systematic Review and Meta-Analysis Protocols

Criteria for relevant articles that investigated the assessment of knowledge, attitude, and perceived barriers regarding CRC screening practices among HCPs followed the PRISMA guidelines (Figure 1) [8].

The initial search identified a total of 337 records. All duplicate records were removed, leaving 190 records (n = 147). After screening the title and abstract, we identified 60 studies. We excluded 39 out of the 60 considered studies for not meeting the study inclusion criteria. Hence, 21 studies were considered eligible and relevant to the research objectives for this review.

## Review

From 2006 to 2023, we identified 21 studies presented in Table 1 that were considered relevant for the current review. Among these studies, eight assess the knowledge, attitude, practices, and perceived barriers toward CRC screening among various health practitioners [9-16]. Three studies assessed health practitioners' knowledge and attitudes toward CRC screening [17-19]. The remaining studies assessed awareness, perceived barriers, or only knowledge of CRC screening among HCPs [20-29]. In addition, all the included studies employed a cross-sectional design.

**Table 1:** Summary of existing literature on the assessment of knowledge, attitude, and perceived barriers regarding CRC screening practices FOBT, fecal occult blood test; SIU, Southern Illinois University; UW, University of Wisconsin; HPRN, High Plains Research Network; HCP, healthcare practitioners; HCWs, healthcare workers; CRC, colorectal cancer

Authors	Title	Country	Study design	Participants characteristics	Characteristics of intervention	Study outcomes
Brown et al. (2015) [9]	CRC screening at community health centres: a survey of clinicians' attitudes, practices, and perceived barriers	USA	Cross-sectional survey	180 clinicians with an average of 11.5 (SD: 9.8) years in practice, 62% were female, and 57% were physicians.	NA	The majority of respondents somewhat agreed (30.2%) or strongly agreed (57.5%) that colonoscopy was the best screening test. However, only 15.8% of respondents strongly agreed and 32.2% somewhat agreed that colonoscopy was readily available for their patients. However, only 24.6% of respondents rated FIT, a type of FOBT, as very effective.
Muliira et al. (2016) [17]	Barriers to CRC screening in primary care settings: attitudes and knowledge of nurses and physicians	Oman	Cross-sectional survey	142 HCPs (57.7% nurses and 42.3% physicians) with an average of 9.39 (SD: 6.13) years in practice.	NA	The results demonstrate that, in spite of their crucial roles in health education, counseling, and the referral of patients who qualify for screening, physicians and nurses working in primary care settings lack sufficient knowledge of CRC screening. Furthermore, there were no significant differences in the attitudes and level of knowledge about CRC screening between nurses and physicians.
Althobaiti, Jradi (2019) [10]	Knowledge, attitude, and perceived barriers regarding CRC screening practices and risk factors among medical students in Saudi Arabia	Saudi Arabia	Cross-sectional survey	581 medical students (47.93% males and 52.07% females) in years four to six in two medical schools.	NA	The findings showed that the students' attitudes regarding CRC screening are poor and their knowledge of CRC risk factors is low.
Birkenfeld, Niv (2006) [20]	Survey of primary physicians’ knowledge of CRC screening	Israel	Cross-sectional survey	133 primary care physicians (family physicians, general practitioners, and others).	NA	Although none of the three groups is familiar with the concept of the high-risk population, the results indicate that family medicine physicians are significantly more knowledgeable about the majority of CRC-related issues than primary care specialists in other fields and general practitioners.
Demyati (2014) [11]	Knowledge, attitude, practice, and perceived barriers to CRC screening among family physicians in National Guard Health Affairs, Riyadh	Saudi Arabia	Cross-sectional survey	130 physicians, consisting of 52.3% females and 47.7% males, with a mean age of 38 years (SD = 7.4). Also, 56.2% of the physicians were not practicing.	NA	Compared to physicians who did not undertake CRC screening, those who reported practicing the procedure had higher knowledge scores. Compared to female physicians, male physicians scored better on the attitude scale. In comparison to practicing physicians, the study indicated that physicians who did not provide CRC screening identified barriers more frequently. The frequently reported barrier was patients' lack of knowledge. Furthermore, despite their level of knowledge and positive attitude, a sizable portion of family physicians do not perform CRC screening.
Sewitch et al. (2006) [12]	CRC screening: physicians’ knowledge of risk assessment and guidelines, practice, and description of barriers and facilitators	Canada	Cross-sectional survey	65 primary care physicians, consisting of 70.8% family medicine physicians and 29.2% general internists. 56.9% were male and 43.1% were female. The mean number of years since graduation from medical school was 21.5 (SD = 8.6).	NA	Although family medicine physicians and general interns showed awareness of the CRC guidelines, they were less knowledgeable in the recommended modalities and periodicities to accurately apply the guidelines to screening patients at an average risk.
Nichols et al. (2009) [13]	Physician knowledge, perceptions of barriers, and patient CRC screening practices	USA	Cross-sectional survey	36 physicians, consisting of family practice physicians (86.2%), male (67.9%), and white (82.3%). Most had been in practice for 16 or more years (60.3%).	NA	The results show that physicians' self-reported screening rates were not optimal, and the majority of physicians stated they had little knowledge of insurance coverage for screening.
Hauer, Wilkerson, Teherani (2008) [21]	The relationship between medical students’ knowledge, confidence, experience, and skills related to CRC screening	USA	Cross-sectional survey	146 third-year medical students in the class of 2007 at the University of California, San Francisco. The study population consisted of 54.4% females and 45.6% males.	NA	The type and amount of experience students had in giving CRC screening counseling predicted their performance in the standardized patient contact, while their knowledge or confidence did not.
Hannon, Martin, Harris, Bowen (2008) [22]	CRC screening practices of primary care physicians in Washington State	USA	Cross-sectional survey	397 physicians (family/general practice = 229, internal medicine = 116, obstetrics/gynecology = 52). The study population consisted of 31.82% females and 68.18% males.	NA	In line with the recommendations of the American Cancer Society, the majority of respondents (76%) suggested one or more CRC tests, and 93% said that patient anxiety around these tests posed a substantial barrier to screening. 90% of physicians said they used the FOBT as a screening test; however, the majority said they did not track their patients' progress or use any kind of incentive to have them finish and return the FOBT kits.
Rim et al. (2009) [14]	Knowledge, attitudes, beliefs, and personal practices regarding CRC screening among healthcare professionals in rural Colorado: a pilot survey	USA	Cross-sectional survey	109 HPRN of rural Colorado prior to a community-based educational intervention. 70% were aged <50 years, and 30% were aged ≥50 years. 63% were family physicians, and most nursing staff professionals were medical assistants (51%).	NA	The results show that a significant percentage of HPRN providers and nursing staff members in general have poor knowledge that CRC is one of the primary causes of cancer death. Furthermore, only 26% of nurses discuss colon cancer prevention and screening with their patients who meet the eligibility criteria; 29% of nurses said that their patients begin the conversation about screening.
Zayegh et al. (2022) [23]	Awareness and knowledge of CRC screening among medical students at the University of Aleppo: a cross-sectional study	Syria	Cross-sectional survey	There were 824 medical students, with females accounting for 52.4% and males accounting for 47.6%. Two-thirds of the students were in the clinical years, while one-third were in the pre-clinical years.	NA	The majority of students (79.8%) knew of CRC and its screening procedures (98.9%). They reported poor knowledge about signs and symptoms (52.6%), protective factors (9.9%), and CRC risk factors (16.5% for non-modifiable factors and 11.7% for modifiable factors). About 31.7% of students were capable of determining the right age to start screening for people who are at average risk.
Lussiez et al. (2021) [24]	CRC screening in Ghana: physicians’ practices and perceived barriers	Ghana	Cross-sectional survey	39 physicians, consisting of males (92.1%), females (7.9%), and 70.3%, were between the ages of 31 and 50 years old. The majority practiced in the field of general surgery (41.7%) and held faculty positions (69.4%). About half of the participants (49%) had been in practice for less than 10 years.	NA	Over 10% of physicians would not advise CRC screening for asymptomatic, average-risk patients who fulfilled the age inclusion requirements outlined in the national guidelines. As a preliminary screening test for CRC, just one physician would suggest FOBT. The absence of test facilities and equipment (28.1%) and inadequate training (18.8%) were the primary barriers to not recommending CRC screening with FOBT. 85% of physicians reported low screening awareness or not considering CRC to be a severe health threat as the two main barriers to screening, followed by high screening costs or insufficient insurance coverage (system level).
Sahin, Aker, Arslan (2017) [25]	Barriers to CRC screening in primary care settings in Turkey	Turkey	Cross-sectional survey	78 primary healthcare providers participated in the study: 49.4% (primary family physicians) and 50.6% (family health professionals). Women constituted 11.5% of all participants. The mean age of primary family physicians and professional experience were 43.64 ± 7.09 and 18.65 ± 7.05 years, respectively, and the mean age of family health professionals were 36.34 ± 6.83 and 14.56 ± 8.04 years, respectively.	NA	86.6% of respondents said they screened patients for CRC. While 29.7% of subjects requested the FOBT at the appropriate intervals, just 6.9% suggested a colonoscopy at the appropriate intervals. Additionally, 60.5% of participants reported not using reminders, while 18.2% of patients were aware that the test used was immunochemical FOBT.
Boehler et al. (2011) [18]	Knowledge and attitudes regarding CRC screening among medical students: a tale of two schools	USA	Cross-sectional survey	383 medical students from the SIU School of Medicine and the UW at Madison.	NA	Between the first-year students and the other years at both schools, there were significant differences in the proportion of correctly answered questions about screening recommendations. Between classes and schools, there was consistency in the attitudes toward CRC screening. However, the majority of students lacked sufficient knowledge about the procedure.
Omran, Barakat, Muliira, Aljadaa (2015) [15]	Knowledge, experiences, and barriers to CRC screening: a survey of healthcare providers working in primary care settings	Jordan	Cross-sectional survey	36 HCPs work in health centers, consisting of nurses (45.8%), physicians (45.3%), and others (7.2%). There were 50.4% females and 49.2% males, with a mean age of 35.37 years (SD = 10.53). Also, most had <5 years of clinical practice experience (37.3%).	NA	30% of the participants were aware of the ideal age to start CRC screening for those at average risk. In general, physicians outscored nurses in terms of their knowledge of CRC screening. Despite having low knowledge of the guidelines for CRC screening, 75.7% of HCPs stated that CRC could be prevented. Also, patients' fear of learning they have cancer, a lack of knowledge about CRC screening tests, a lack of qualified healthcare professionals to perform invasive screening procedures, and a lack of resources were the most reported perceived barriers to CRC screening.
Muliira, D'Souza, Ahmed (2016) [26]	Contrasts in practices and perceived barriers to CRC screening by nurses and physicians working in primary care settings in Oman	Jordan	Cross-sectional survey	142 HCPs. The HCPs were nurses (57.1%) and physicians (42.3%), with an average age and clinical experience of 32.5 and 9.5 years, respectively. There were 92.3% females and 7.7% males, with a mean age of 32.5 years (SD = 6.5).	NA	Among eligible patients, the majority of HCPs (64.8%) seldom ordered, referred, health-educated, or suggested CRC screening. HCPs noted that the only significant patient-related barrier to CRC screening was poor knowledge of CRC testing (63.7%). The absence of hospital policies or procedures, a shortage of qualified healthcare professionals, a lack of CRC screening services, and a lack of timely appointments for CRC screening were the main institutional barriers to CRC screening.
Şahin and Aker (2017) [16]	Family physicians’ knowledge, attitudes, and practices toward CRC screening	Turkey	Cross-sectional survey	290 family physicians consisting of males (65.6%) and females (34.1%), with a median age of 43.40 years (SD = 6.54). The median working experience was 18.43 years (SD = 6.42).	NA	83.1% of respondents conducted CRC screenings, 37.7% advised FOBT at the appropriate frequency, and 11.7% advocated colonoscopy. A further 68.6% of doctors did not adhere to any CRC guidelines. Of those who reported utilizing a guideline, just 3.8% could identify it. In this study, the vast majority performed CRC screening. The respondents do not have sufficient knowledge of the ages at which screening tests should begin and end, as well as the frequency at which they should be conducted. They also fail to assign the CRC guidelines enough importance.
Mosli et al. (2017) [19]	Knowledge, attitude, and practices of primary healthcare physicians toward CRC screening	Saudi Arabia	Cross-sectional survey	127 primary healthcare physicians consisting of females (56.7%) and males (47.3%), with a mean age of 34 years (SD = 8.49).	NA	Even though most primary care physicians agree that CRC screening is beneficial, a significant percentage of them disregard the recommendations. Lower-qualified male primary care providers seem to be less inclined to suggest screening.
Ooi, Hanafi, Liew (2019) [27]	Knowledge and practice of CRC screening in an urban setting: a cross-sectional survey of primary care physicians in government clinics in Malaysia	Malaysia	Cross-sectional survey	197 primary care physicians consisted of females (78.2%) and males (21.8%), with a mean age of 35.4 years (SD = 7.6).	NA	Primary care physicians lacked sufficient knowledge and expertise in CRC screening. The practice of screening did not follow from knowledge of it. Their opinions regarding the cost-effectiveness of the process and the availability of sufficient resources significantly influenced the practice of screening.
Papanikolaou et al. (2012) [28]	Awareness and attitudes of Greek medical students on CRC screening	Greece	Cross-sectional survey	260 medical students consisted of females (47%) and males (53%), with a mean age of 22.8 years (range: 21–25 years).	NA	Merely 69% of the participants in the study believed that CRC posed a major risk to public health. Nonetheless, the overwhelming majority of participants recognized symptoms associated with CRC and agreed that screening for the disease is crucial for lowering its incidence and mortality. Merely 38% of the participants reported having come across information about CRC screening, either as part of their medical education or as part of public education. Additionally, only 60% of the participants indicated that they would be open to receiving more information. In terms of colonoscopy, 85% would rather use a different procedure for screening for CRC. Furthermore, 68% of respondents said they would like more information about the procedure, and 53% said it was an unpleasant procedure.
Magwaza, Van Hal, Hoque (2023) [29]	Knowledge, attitude, and practices of healthcare workers towards CRC screening in primary care settings in Durban, South Africa: a cross-sectional survey	South Africa	Cross-sectional survey	109 HCWs in public primary healthcare facilities consist of females (88%) and males (12%). The majority were between the ages of 30 and 39 years old (33%) and had more than 10 years of working experience (47%).	NA	The great majority of healthcare workers were poorly knowledgeable of the CRC screening program's protocols. Nonetheless, once trained, the great majority of HCWs were willing to perform screening.

Among the included studies, six were conducted in the USA, three in Saudi Arabia, two in Jordan and Turkey, and one in Canada, Ghana, Greece, Israel, Malaysia, Oman, South Africa, and Syria. The sample size of the included studies ranges from 36 to 581. Furthermore, 11 included studies were carried out among physicians, including family physicians, primary care physicians, and general physicians. Five studies used general healthcare workers, including doctors and nurses. The remaining five studies used medical students. All of these things are clarified in Table 1.

Discussion

Globally, CRC is the second leading type of cancer in women and the third leading type in males [1]. CRC screening significantly reduces both the incidence and death of CRC cancers [2, 18]. Researchers have indicated that physician advice is the most significant predictor of patient compliance with CRC screening [2, 7]. The present review presents a comprehensive literature review on the level of knowledge, attitude, practices, and perceived barriers toward CRC screening among health practitioners. Most of the studies investigated the level of knowledge, attitude, and practice of CRC screening among physicians. It is critical to comprehend the healthcare providers' viewpoint on CRC screening, given the significance of early screening, especially when advised by healthcare providers.

The findings of this review illustrate that a high proportion of HCPs need to gain more knowledge about CRC screening. For example, many healthcare providers need to gain current knowledge regarding CRC screening and the guidelines for widely used CRC screening techniques, including FOBT, sigmoidoscopy, and colonoscopy [15, 26, 28]. Most medical professionals stated that their knowledge of CRC screening and prevention needed to be improved. Studies focusing primarily on medical professionals have revealed similar results, demonstrating that most medical students graduate without the skills required to help patients prevent and detect cancer and that numerous physicians lack adequate training and confidence in essential cancer prevention and detection techniques [18, 22, 29].

Our study's findings demonstrate that most healthcare providers needed better CRC screening practices since they infrequently carried out tasks, including ordering, referring, educating patients about their health, or suggesting CRC screening for eligible patients [10, 11, 13, 17]. Healthcare professionals' CRC screening practices were impacted by the absence of mechanisms to identify eligible individuals, the presence of cancer experts, continuing professional education on cancer prevention, and cancer screening policies at health facilities [13, 15]. Raising the level of knowledge among healthcare workers through ongoing education initiatives and establishing specific regulations, procedures, and guidelines for CRC screening inside healthcare facilities are likely to improve CRC screening [15]. Research shows that medical students' professional habits and practices greatly depend on the knowledge and attitudes they acquire in medical school or professional training programs [15, 18].

The most reported barriers to CRC screening are a lack of qualified personnel to follow-up with CRC screening techniques and patients' anxiety over learning they have cancer [9, 12, 15, 23]. Health professionals' knowledge and inability to inform patients about the advantages of CRC screening, available screening test alternatives, potential side effects, and the specifics of the procedures could exacerbate these barriers [15, 23]. The health professionals also mentioned the lack of qualified healthcare professionals and patient follow-up, the cost, the accessibility of screening services, the absence of a policy, the length of waiting periods for appointments, and the volume of patients [15, 23]. In addition, primary care providers expressed their top concerns about colonoscopy screening, including a lack of information, time constraints, and patient anxiety over the invasive nature of the procedure, and patients' perceptions of FOBT's benefits [12].

This article mainly focuses on the level of knowledge, attitude, practices, and perceived barriers toward CRC screening among health practitioners. However, the study does have limitations despite being conducted across various parts of the world. First, all the studies were conducted using a cross-sectional survey design; this illustrates the need to develop appropriate interventions to improve knowledge, attitude, practice, and perceived barriers and test their efficacy among HCPs. Second, only the studies limited to HCPs were included in the current study. Future studies could consider reviewing studies involving different demographics, such as patients and healthy people.

## Conclusions

Research has demonstrated that doctors play a significant role in raising the low rates of CRC screening. However, there is a need for further improvement in healthcare practices to enhance healthcare professionals' knowledge, attitudes, and practices regarding CRC screening and their perception of its barriers. It emphasizes how critical it is that healthcare providers pay more attention to their critical role in CRC screening, both in their professions and education.
